# A repeated cross-sectional survey assessing university students' stress, depression, anxiety, and suicidality in the early stages of the COVID-19 pandemic in Poland

**DOI:** 10.1017/S003329172000392X

**Published:** 2020-10-02

**Authors:** Agata Debowska, Beata Horeczy, Daniel Boduszek, Dariusz Dolinski

**Affiliations:** 1 Department of Psychology, The University of Sheffield, Sheffield, UK; 2 Faculty of Psychology and Law, SWPS University of Social Sciences and Humanities, Poznan, Poland; 3 Anesthesiology and Intensive Care Department with the Center for Acute Poisoning, St. Jadwiga Provincial Clinical Hospital, Rzeszow, Poland; 4 Medical College, University of Rzeszow, Rzeszow, Poland; 5 Faculty of Psychology, SWPS University of Social Sciences and Humanities, Katowice, Poland; 6 Department of Psychology, University of Huddersfield, Huddersfield, UK; 7 Faculty of Psychology, SWPS University of Social Sciences and Humanities, Wroclaw, Poland

**Keywords:** Anxiety, COVID-19 pandemic, depression, stress, suicidality, university students

## Abstract

**Background:**

The time of widespread outbreaks of infectious diseases can lead to elevated stress and mental health problems among all persons affected, and in particular those sub-groups of the population that are at an increased risk of mental health problems. One such vulnerable group constitutes university students. The aim of this study is to assess stress, depression, anxiety, and suicidality among different groups of university students (medical, psychology, and other).

**Methods:**

Using a repeated cross-sectional study design, we collected survey data among a large sample of 7228 university students from Poland (mean age = 22.78, s.d. = 4.40; 81% female). Data were collected in five waves, during the first 2 months of the COVID-19 pandemic in Europe (March and April 2020).

**Results:**

The results demonstrate a significant increase in depression levels as the pandemic was progressing. We also found that female students scored significantly higher than male students on depression, anxiety, and stress. Psychology students recorded the lowest scores on depression and anxiety. Young adult students (aged 18–24 years) had more symptoms of depression, anxiety, and suicidality than adult students (⩾25 years).

**Conclusions:**

These results provide insights into stress and mental health among university students during the early stages of the COVID-19 pandemic. Findings can be used for a more effective identification of students who may struggle during next stages of the pandemic and future crises.

The time of the COVID-19 pandemic can be emotionally challenging and stressful to all persons affected, and in particular those sub-groups of the population that are at an increased risk of mental health problems. One such vulnerable group constitutes university students (Wang et al., 2020).

Using a repeated cross-sectional study design, we assessed stress, depression, anxiety, and suicidality among medical, psychology, and other students (*N* = 7228, 81% female; *M* age = 22.78, s.d. = 4.40). Participants were recruited via 10 Polish universities and the Students' Parliament of the Republic of Poland. Data collection occurred in five stages, during the first 2 months of the COVID-19 pandemic in Europe (March–April 2020). The stages differed from one another in the amount and type of lockdown-type measures, with stage 4 being characterised by the strictest restrictions (see note in Table 1).

**Table 1.** Descriptive statistics including means (*M*) and standard deviations (s.d.) for suicidality, depression, anxiety, and stress across the study stages and genders

	Stage 1	Stage 2	Stage 3	Stage 4	Stage 5
*Suicidality*					
Males					
*M*	1.27	1.09	1.37	1.53	1.64
s.d.	2.02	1.60	2.10	2.27	2.41
*N*	90	35	635	434	188
Females					
*M*	1.28	1.37	1.31	1.32	1.23
s.d.	2.11	2.13	2.08	2.14	2.01
*N*	454	256	2715	1577	844
Total					
*M*	1.27	1.33	1.32	1.36	1.30
s.d.	2.09	2.07	2.08	2.17	2.09
*N*	544	291	3350	2011	1032
*Depression*					
Males					
*M*	12.11	12.76	12.63	13.24	12.93
s.d.	9.69	10.08	10.27	10.75	10.44
*N*	87	34	588	401	174
Females					
*M*	12.18	13.82	15.05	15.57	14.53
s.d.	10.06	10.41	10.86	11.08	10.48
*N*	442	255	2548	1459	806
Total					
*M*	12.17	13.70	14.59	15.07	14.25
s.d.	9.99	10.36	10.79	11.05	10.49
*N*	529	289	3136	1860	980
*Anxiety*					
Males					
*M*	7.06	6.12	6.48	6.58	7.00
s.d.	7.10	8.25	7.59	7.34	8.19
*N*	87	34	590	402	174
Females					
*M*	10.01	9.36	9.83	10.44	9.39
s.d.	9.10	9.45	9.17	9.69	9.09
*N*	443	255	2547	1461	808
Total					
*M*	9.52	8.98	9.20	9.61	8.96
s.d.	8.86	9.39	8.99	9.37	9.98
*N*	530	289	3137	1863	982
*Stress*					
Males					
*M*	14.80	15.12	13.25	13.80	14.40
s.d.	10.16	10.51	9.76	10.69	10.77
*N*	87	34	589	403	174
Females					
*M*	16.95	17.61	17.88	18.62	18.43
s.d.	10.16	10.53	10.58	11.04	10.45
*N*	442	255	2554	1462	807
Total					
*M*	16.60	17.31	17.01	17.57	17.71
s.d.	10.18	10.54	10.58	11.14	10.61
*N*	529	289	3143	1865	981

*Note*: Stage 1 = data collected 1–15 March (first COVID-19 infections in Poland were announced; no lockdown-type measures); stage 2 = data collected 16–22 March (first lockdown-type control measures, including closing schools and university classes and cancelling mass events); stage 3 = data collected 23–29 March (strengthening of lockdown-type measures, limiting non-family gatherings to two people and forbidding non-essential travel); stage 4 = data collected 30 March–5 April (another tightening of lockdown restrictions occurred, requiring individuals in the streets to be separated by 2 m, closing parks, boulevards, beaches, hairdressers and beauty salons, and forbidding unaccompanied minors from exiting their homes); stage 5 = data collected 6–30 April (all individuals were required to wear face coverings in public spaces; the restriction on public gatherings was loosened).

Depression, anxiety, and stress levels in the past week were measured using the Depression Anxiety Stress Scales (DASS; Lovibond and Lovibond, 1995). The frequency and intensity of suicidal ideation and impulses in the past 24 h were measured using the Depressive Symptom Inventory-Suicidality Subscale (DSI-SS; Joiner, Pfaff, and Acres, 2002).

We performed 2 (gender) × 5 (study stage) between group analyses of variance (ANOVAs) to explore the differences between males and females and five stages of data collection on suicidality, depression, anxiety, and stress (Table 1). Our findings demonstrate a significant increase in depression as the pandemic was progressing. Specifically, depression scores in study stages 3, 4, and 5 increased significantly from stage 1. Stages 3, 4, and 5 differed from stage 1 in that lockdown-type measures mostly relying on social distancing and isolation were introduced to control the spread of the disease (Fig. 1). Loneliness was previously found to contribute to the development of depressive symptoms (Grygiel et al., 2013). The present findings, therefore, may indicate that because students had to isolate to avoid infection, their desire for social connectedness was not met, resulting in more symptoms of depression.

**Fig. 1.**
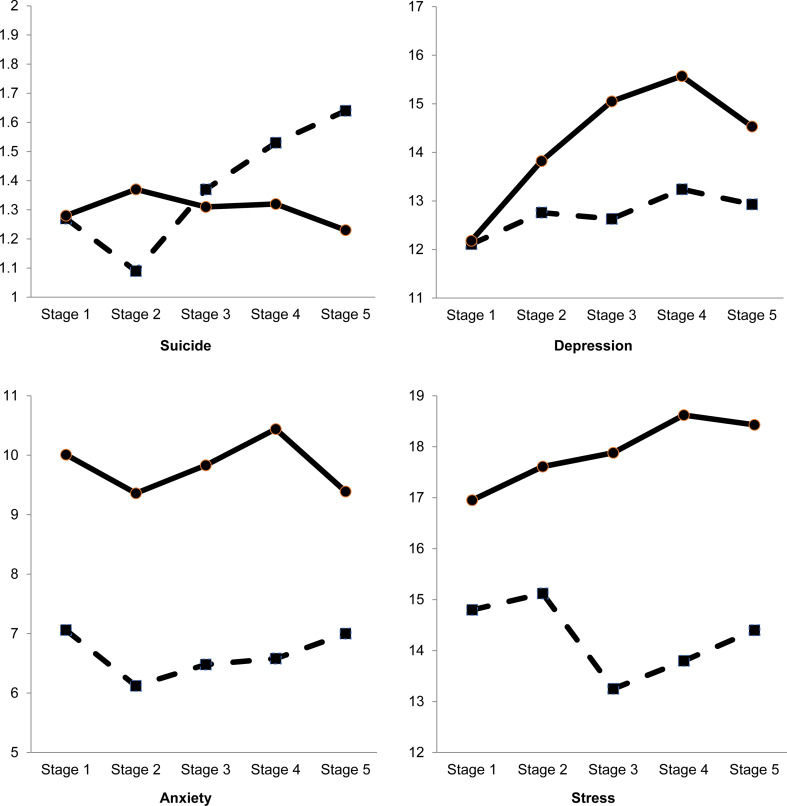
A 2 (gender) by 5 (study stages) ANOVA results. *Note*: Solid line = females; dashed line = males. 2 (gender) × 5 (study stage) ANOVAs results indicated no statistically significant interaction between gender and study stage for all variables. With regards to suicidality, there was no significant main effect for gender or study stage. There was a statistically significant main effect for gender (*F*_(1, 6784)_ = 8.21, *p* = 0.004, *η*^2^ = 0.001) and study stage (*F*_(4, 6784)_ = 2.77, *p* = 0.03, *η*^2^ = 0.002) on depression scores. Post-hoc Tukey's HSD test indicated significant differences between stages 1 and 3 (*p* < 0.001; Cohen's *d* = 0.23), stages 1 and 4 (*p* < 0.001; Cohen's *d* = 0.28), as well as stages 1 and 5 (*p* = 0.003; Cohen's *d* = 0.20). There was a statistically significant main effect for gender on anxiety (*F*_(1, 6791)_ = 51.78, *p* = 0.000, *η*^2^ = 0.008) and stress (*F*_(1, 6797)_ = 49.43, *p* < 0.001, *η*^2^ = 0.007). No significant effect for study stage was recorded on anxiety or stress.

We also found that female students scored significantly higher than male students on depression, anxiety, and stress. Anxiety and stress levels were higher among women at all study stages, including stage 1, which could indicate that these gender differences were present before the pandemic. Indeed, there is substantial empirical evidence collected at a time when there was no crisis indicating that women, compared with men, report higher levels of anxiety and stress (Gentry et al., 2007; McLean & Anderson, 2009). Interestingly, Anderson and Manuel (1994) found that women experienced greater amounts of stress in response to an earthquake than men. Therefore, it seems that gender differences in stress and anxiety precede the pandemic, but the pandemic may deepen this discrepancy.

As for depression, men and women reported a similar number of symptoms at stage 1, but there was a substantial gender difference in depression scores in the subsequent study stages, with women experiencing more symptoms. Although both genders recorded an increase in the symptoms of depression as the pandemic was progressing, the increase was more pronounced in females. Our finding highlights a potential pandemic effect on the emergence of gender differences in depression. We theorise that this may be due to increased caring responsibilities, more worry about the well-being of family and friends, or an unmet need for social connectedness, all of which are more likely to affect women than men.

As for differences between student groups, we found that medical (*p* = 0.016; Cohen's *d* = 0.12) and other students (*p* < 0.001; Cohen's *d* = 0.22) scored higher than psychology students on depression. With regards to anxiety, other students scored higher than psychology students (*p* = 0.049; Cohen's *d* = 0.07). There were no significant differences between the groups on stress and suicidality. These findings may indicate staff and students at psychology departments are more aware of the potential impact of the pandemic on mental health. Thus, staff may offer more support to students and students may create mutual support groups.

We also found that young adult students (18–24 years) scored significantly higher than adult students (⩾25 years) on suicidality (*p* < 0.001, Cohen's *d* = 0.25), depression (*p* < 0.001, Cohen's *d* = 0.19), and anxiety (*p* < 0.001, Cohen's *d* = 0.15), which confirms the theoretical considerations suggesting that young adult students who are in the processes of achieving important developmental milestones and, at the same time, face stressors associated with academic studies, are particularly prone to psychological distress (Arnett, 2004; Dusselier, Dunn, Wang, Shelley, & Whalen, 2005).

Although this study is not free from limitations, our findings provide insights into stress and mental health among university students during the early stages of the COVID-19 pandemic. Findings can be used for a more effective identification of students who may struggle during next stages of the pandemic and future crises.
